# Integrating MRI habitat heterogeneity and peritumoral radiomics into a nomogram for optimized risk stratification in locally advanced rectal cancer: a multicenter study

**DOI:** 10.3389/fonc.2026.1739343

**Published:** 2026-04-21

**Authors:** Liang Zhang, Jizheng Lin, Dacheng Li, Guohua Wang, Xueting Qu

**Affiliations:** 1 Nuclear Medicine Department, The Affiliated Hospital of Qingdao University, Qingdao, Shandong, China; 2 Radiology Department, Qingdao Municipal Hospital, Qingdao University, Qingdao, Shandong, China; 3 Radiology Department, The Affiliated Hospital of Qingdao University, Qingdao, Shandong, China; 4 Radiology Department, Qingdao Traditional Chinese Medicine Hospital, Qingdao Hiser Hospital Affiliated of Qingdao University, Qingdao, Shandong, China

**Keywords:** Habitat, MRI, radiomics, rectal cancer, risk assessment

## Abstract

**Background:**

Risk stratification is essential for optimizing treatment in locally advanced rectal cancer (LARC), particularly for identifying suitable candidates for total neoadjuvant therapy (TNT). Conventional MRI-based staging has limited sensitivity in capturing tumor microenvironment (TME) heterogeneity, which may lead to undertreatment of high-risk patients or overtreatment of low-risk subgroups. This study aimed to develop a nomogram integrating MRI-based habitat heterogeneity and peritumoral radiomics to improve risk stratification in LARC.

**Methods:**

A multicenter retrospective cohort of 290 LARC patients (training set, n=178; external test set, n=112) was analyzed. Tumor volumes and peritumoral regions (1, 2, and 3 mm margins) were delineated on high-resolution MRI scans using 3D Slicer. Habitat heterogeneity was quantified via K-means clustering (k=3) of intratumoral radiomic features. Radiomic features were filtered and reduced using LASSO regression. Logistic regression (LR) and support vector machine (SVM) classifiers were used to build intratumoral, peritumoral, and habitat models. The better-performing model between the intratumoral and habitat models was combined with the optimal peritumoral model and clinical variables to construct a nomogram. Calibration curves assessed agreement between predicted and observed high-risk LARC. Model performance was evaluated using area under the curve (AUC), sensitivity, specificity, and decision curve analysis (DCA).

**Results:**

LR classifiers outperformed SVM classifiers and were therefore selected for the intratumoral, peritumoral, and habitat models. The habitat and peritumoral (3 mm) models showed superior performance compared with the intratumoral and peritumoral (1 mm) models and were integrated with clinical variables into a nomogram. The nomogram achieved excellent performance in both training (AUC, 0.928) and test cohorts (AUC, 0.817), surpassing single-feature models. Calibration curves demonstrated good agreement between predicted and observed high-risk LARC. DCA showed the nomogram provided higher net benefit across a broad range of threshold probabilities.

**Conclusion:**

By characterizing the spatial heterogeneity of the tumor microenvironment, an MRI-derived nomogram integrating habitat heterogeneity, peritumoral (3 mm) radiomic features, and clinical variables was developed to facilitate precise risk stratification and personalized TNT decision-making for LARC.

## Introduction

Locally advanced rectal cancer (LARC) presents substantial challenges in clinical management owing to its high recurrence rate and considerable tumor heterogeneity (1, 2). In 2022, there were over 1.9 million new cases of colorectal cancer and approximately 900,000 deaths worldwide (3). Among patients with LARC, the postoperative recurrence rate was 23%, while the 3-year recurrence-free survival rate was 76% (4). Currently, the standard treatment protocol consists of neoadjuvant chemoradiotherapy (nCRT) followed by total mesorectal excision. Nevertheless, roughly 30% of high-risk patients still experience treatment resistance or develop distant metastasis (5–7).

In recent years, total neoadjuvant therapy (TNT), which combines upfront systemic chemotherapy with radiotherapy, has shown advantages such as improved treatment compliance, early management of micrometastases, and higher rates of pathological complete response (pCR) (8, 9). Phase III clinical trials, including RAPIDO and PRODIGE 23, have validated the survival benefits of TNT over nCRT (8, 10). However, the intensified TNT regimen may result in overtreatment among low-risk patients (e.g., those with cT3N0 disease and negative mesorectal fascia [MRF]), leading to increased toxicity without a corresponding survival advantage (11). Consequently, the National Comprehensive Cancer Network (NCCN) guidelines stress the importance of accurately identifying high-risk LARC patients to optimize TNT application (12).

Clinical risk stratification currently relies mainly on TNM staging and MRI assessment. However, conventional MRI has difficulty resolving the spatial heterogeneity of the tumor microenvironment (TME) (13). Research demonstrates that TME characteristics—such as immune cell infiltration, stromal fibrosis, and angiogenesis—are strongly linked to treatment response, yet these microscopic heterogeneities cannot be quantified using conventional imaging techniques (14, 15). Radiomics, which enables high-throughput extraction of imaging features, provides a novel approach for analyzing tumor heterogeneity. Nevertheless, its inherent limitation, based on the assumption of a “homogeneous tumor,” is becoming increasingly evident (15–17). Habitat analysis, which segments intratumoral heterogeneous subregions and deciphers their spatial interactions, offers a potential noninvasive method for decoding the TME (18). For example, Shi et al. (19) demonstrated that MRI-based habitat radiomics effectively predicts the development of metachronous liver metastasis in patients with LARC, while Li et al. (20) showed that habitat imaging in breast cancer accurately identifies biologically heterogeneous tumor regions, facilitating assessment of neoadjuvant chemotherapy response. However, current studies often focus on single modalities or single-scale features, lacking multidimensional models that integrate intratumoral heterogeneity, peritumoral microenvironment, and clinical parameters.

This study seeks to overcome the dimensional constraints of traditional imaging evaluation by developing an innovative MRI-based habitat analysis model. By integrating intratumoral heterogeneous subregions, the peritumoral microenvironment, and clinical characteristics, we establish a predictive model for patients with high-risk LARC. The expected outcomes aim to identify imaging biomarkers for the precise selection of TNT candidates, facilitating the shift in LARC management from a uniform approach to individualized stratification.

## Materials and methods

### Study design and patient population

Clinical and MRI data were retrospectively gathered from rectal cancer patients who underwent surgical resection at four hospitals. Patients from one hospital comprised the training cohort, while those from the remaining three hospitals formed the external test cohort (details are provided in **Supplementary Material S1**). Inclusion criteria were: (1) pathologically confirmed rectal adenocarcinoma following radical resection; (2) preoperative MRI examination conducted within two weeks prior to surgery. Exclusion criteria included: incomplete clinicopathological data, incomplete or poor-quality MRI scans, the presence of distant metastasis, and receipt of any neoadjuvant therapy prior to surgery.

### Clinical and imaging variables

Collected clinical and imaging characteristics included: Age, Sex, Carcinoembryonic Antigen (CEA), Carbohydrate Antigen 199 (CA199), Distance from anal verge (DIS), Depth of invasion, Tumor length, mrT stage, mrN stage, Circumferential growth status (Cir), Mesorectal fascia (MRF), and Extramural Venous Invasion (EMVI).

### Risk stratification

**Figure 1:**
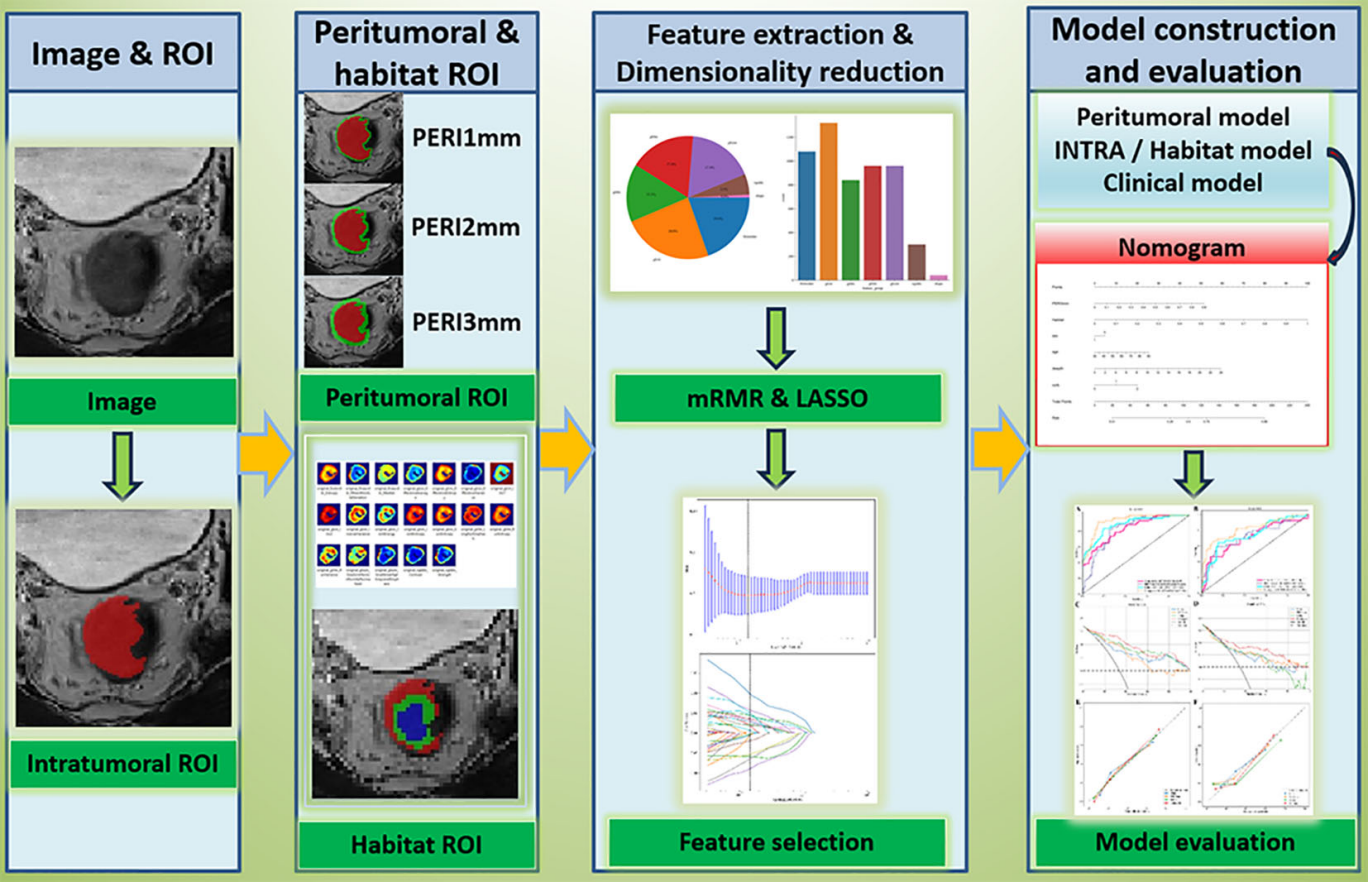
The flow chart of this study.

Patients were stratified into high-risk and low-risk groups based on postoperative pathological staging according to NCCN guidelines. In this study, high-risk LARC was defined by any of the following based on postoperative pathology: T4, N2, lateral lymph node metastasis, or mesorectal fascia involvement. All enrolled patients underwent radical surgery; postoperative pathological staging was performed to record these parameters, and patients meeting any criterion were assigned to the high-risk group. The study flowchart is illustrated in **Figure 1**.

### MRI image acquisition and preprocessing

All participating centers employed 3.0T MRI scanners. Patients underwent scanning in the supine position, without specific bowel preparation. Detailed scanning parameters are provided in **Supplementary Material S1**. Original axial T2-weighted images (T2WI) were imported into 3D Slicer software (version 5.0.3). To ensure consistency across multicenter data, the following preprocessing steps were implemented: (1) Intensity inhomogeneity artifacts were corrected using the N4 algorithm. (2) Z-score normalization was applied to the entire dataset to reduce inter-scanner signal variability. (3) Anisotropic images were resampled to isotropic voxels (1 × 1 × 1 mm³) via third-order B-spline interpolation to maintain textural feature continuity.

### Intratumoral volume of interest generation

Two abdominal radiologists, with 6 and 15 years of experience respectively, manually delineated the tumor boundary slice by slice on axial T2-weighted imaging (T2WI) using 3D Slicer (version 5.0.3). The segmentation included the entire tumor region while excluding intraluminal gas, thereby generating the intratumoral VOI (VOI_INTRA). A random sample of 30 cases underwent re-segmentation by the same radiologist after a 30-day interval to assess intra-observer consistency and by the second radiologist independently to evaluate inter-observer consistency. Intra-class and inter-class correlation coefficients (ICCs) were computed. Only features with ICCs ≥ 0.75 were retained to ensure robustness.

### Peritumoral VOI generation

A morphological dilation operation, based on VOI_INTRA and executed using Onekey AI software, was applied to achieve three-dimensional expansion outward by distances of 1 mm, 2 mm, and 3 mm. Regions that overlapped with the bowel lumen were manually removed. The resulting peritumoral VOI were labeled as VOI_PERI1mm, VOI_PERI2mm, and VOI_PERI3mm.

### Habitat VOI generation

Voxel-wise Feature Extraction: VOI_INTRA was resampled to 2 mm³ voxels. Using PyRadiomics (http://pyradiomics.readthedocs.io), 19 radiomic features were extracted for each voxel within VOI_INTRA (**Supplementary Material S2**).

**Figure 2:**
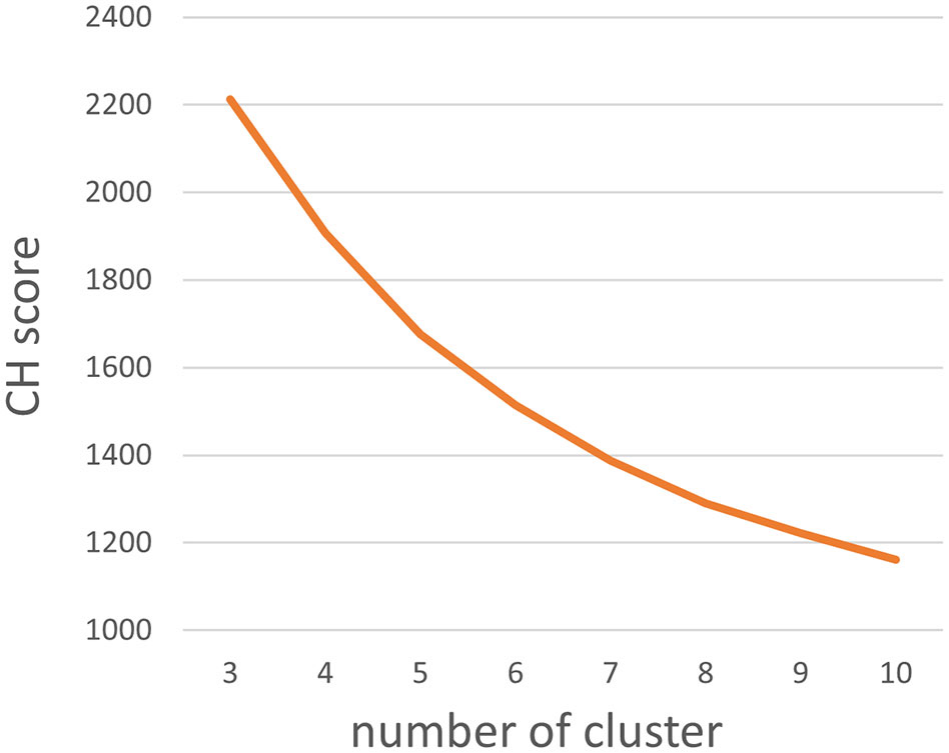
The CH score with different number of clusters.

**Figure 3:**
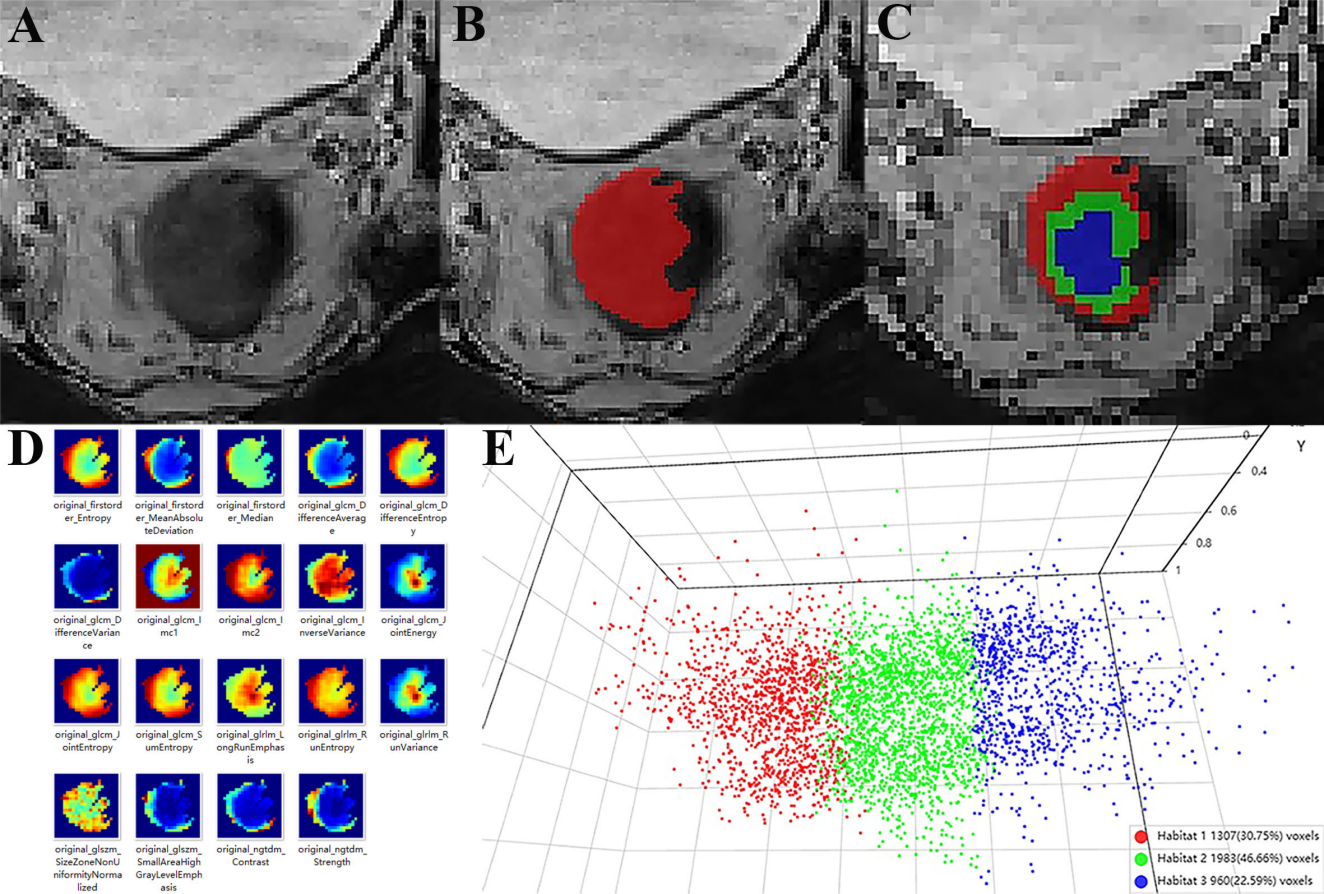
Intratumoral subregion segmentation. The T2WI figure of the tumor **(A)**, intratumoral ROI **(B)**, three different habitat subregion **(C)**, lesion voxel radiomics feature extraction **(D)**, 3D visualization projection of each voxel radiomics feature **(E)**.

Clustering Analysis: K-means clustering (implemented via scikit-learn library) was performed with cluster numbers ranging from n=3 to n=10. The optimal number of clusters was determined as n=3 based on the maximum Calinski-Harabasz (CH) index value (**Figure 2**). The three resulting habitat subregions were labeled VOI_h1, VOI_h2, and VOI_h3 (**Figure 3**).

### Feature extraction

Prior to extraction, images were Z−score normalized, multiplied by a scaling factor of 100, shifted by 300 voxel intensity to ensure positive intensities, and discretized with a bin width of 5, after resampling to an isotropic voxel size using B−spline interpolation. Features were extracted from the original images, Laplacian of Gaussian (LoG) filtered images (sigma = 2, 3, 4, 5 mm), and wavelet-transformed images. Gray-level discretization was performed with a fixed bin width of 5. Feature classes included shape, first-order statistics, GLCM, GLRLM, GLSZM, GLDM, and NGTDM. For GLCM. The following VOIs were used for feature extraction: (1) Intratumoral VOI: feature_INTRA (1197 features); (2) Peritumoral VOIs: feature_PERI1mm, feature_PERI2mm, feature_PERI3mm (each with 1197 features); (3) Habitat VOIs: feature_h1, feature_h2, feature_h3 (each with 1197 features). Features from the three habitat subregions were concatenated to form feature_habitat (3591 features).

### Feature selection and dimensionality reduction

All radiomic features, including intratumoral, peritumoral, and habitat features, were standardized using Z-score normalization. Subsequently, the data were scaled to the range of 0 to 1 to promote balance, reduce overfitting, and improve model generalizability. Features with intra-ICCs or inter-ICCs below 0.75 were excluded. Statistical comparisons were performed using the Mann-Whitney U test for non-normally distributed data and the independent samples t-test for normally distributed data. Features with p-values ≥ 0.05 were removed. The Spearman rank correlation coefficient (ρ) was computed for each pair of features, and when ρ exceeded 0.9, only one feature from the pair was retained. The maximum Relevance Minimum Redundancy (mRMR) algorithm was then used to select the 40 most informative and least redundant features, balancing informational value against the risk of overfitting. Finally, Least Absolute Shrinkage and Selection Operator (LASSO) regression with 10-fold cross-validation was applied to the mRMR-selected features, with the goal of minimizing binomial deviance. Features with non-zero coefficients were retained to construct the Radiomics Score (RadScore). All feature selection steps, including mRMR and LASSO with 10-fold cross-validation, were performed exclusively within the training cohort. To prevent information leakage, the LASSO feature selection was nested inside the cross-validation loop, ensuring that feature selection was independently performed within each training fold.

### Predictive model construction

Models were built using Logistic Regression (LR) and Support Vector Machine (SVM) classifiers.

Radiomics Models: Filtered features were input into classifiers to build separate models: Intratumoral Model (INTRA), Peritumoral Models (PERI1mm, PERI2mm, PERI3mm), Habitat Model (Habitat).

Clinical Model: Univariate and multivariate Logistic Regression analyses were performed to identify clinical risk factors significantly associated with high-risk LARC. A clinical prediction model (Clinical) was constructed using these factors.

Integrated Model (Nomogram): The most predictive radiomics model (either intratumoral or habitat), the best-performing peritumoral model, and the clinical model were integrated to construct an integrated model. A visualized nomogram was constructed.

### Model evaluation and statistical analysis

Model performance was evaluated using metrics including the Area Under the Receiver Operating Characteristic Curve (AUC), accuracy, sensitivity, specificity, Positive Predictive Value (PPV), and Negative Predictive Value (NPV). Calibration was assessed using calibration curves and the Hosmer-Lemeshow test to evaluate the agreement between predicted and observed probabilities. Decision Curve Analysis (DCA) was conducted to quantify the net clinical benefit of the models across a range of threshold probabilities for predicting high-risk LARC.

### Statistical tests

Python 3.8 (http://www.python.org) was used to statistical analyses. The Kolmogorov-Smirnov test was used to assess normality and variance homogeneity. Employed t-test, Mann-Whitney U test, or Chi-square test as appropriate were performed to group comparisons and feature screening. Utilized univariate and multivariate Logistic Regression were used to select the clinical risk factors. DeLong test was used to compare AUCs between the models. The significance level was set at p < 0.05.

## Results

### Patient cohort characteristics

This study ultimately included 290 LARC patients (Training cohort: n=178; External test cohort: n=112). The high-risk group accounted for 33.1% (99/290) of the total cohort. Baseline characteristics of the multicenter cohorts are presented in **Table 1**. Compared to the low-risk group, the high-risk group exhibited significantly longer tumor length, greater depth of invasion, a higher proportion of circumferential growth, higher rates of MRF positivity and EMVI positivity, and significantly higher proportions of mrT3–4 and mrN2 stages.

**Table 1:** Clinical baseline characteristics of training and test cohorts.

Feature	Training		P value	Test		P value
	Low-risk (n=118)	High-risk (n=60)		Low-risk (n=73)	High-risk (n=39)	
age	63.69 ± 9.93	64.02 ± 11.68	0.784	63.34 ± 9.49	64.28 ± 8.76	0.609
DIS	76.18 ± 31.85	78.65 ± 32.41	0.608	71.60 ± 30.55	76.79 ± 33.42	0.409
length	41.01 ± 13.43	49.38 ± 12.14	<0.001	40.86 ± 11.62	47.38 ± 14.11	0.009
depth	1.76 ± 2.46	6.92 ± 6.43	<0.001	1.93 ± 3.06	3.85 ± 3.30	<0.001
sex			0.091			0.376
Female	42(35.59)	30(50.00)		26(35.62)	18(46.15)	
Men	76(64.41)	30(50.00)		47(64.38)	21(53.85)	
Cir			<0.001			0.036
No	65(55.08)	15(25.00)		39(53.42)	12(30.77)	
Yes	53(44.92)	45(75.00)		34(46.58)	27(69.23)	
mrT			<0.001			<0.001
T1	8(6.78)	1(1.67)		1(1.37)	null	
T2	25(21.19)	4(6.67)		26(35.62)	5(12.82)	
T3	83(70.34)	44(73.33)		45(61.64)	26(66.67)	
T4	2(1.69)	11(18.33)		1(1.37)	8(20.51)	
mrN			<0.001			0.01
N0	82(69.49)	17(28.33)		32(43.84)	10(25.64)	
N1	31(26.27)	18(30.00)		33(45.21)	16(41.03)	
N2	5(4.24)	25(41.67)		8(10.96)	13(33.33)	
MRF			<0.001			<0.001
Negative	118(100.00)	34(56.67)		73(100.00)	21(53.85)	
Positive	null	26(43.33)		null	18(46.15)	
EMVI			<0.001			<0.001
Negative	108(91.53)	41(68.33)		72(98.63)	24(61.54)	
Positive	10(8.47)	19(31.67)		1(1.37)	15(38.46)	
CEA			<0.001			0.092
Negative	88(74.58)	24(40.00)		54(73.97)	22(56.41)	
Positive	30(25.42)	36(60.00)		19(26.03)	17(43.59)	
CA199			0.197			0.582
Negative	112(94.92)	53(88.33)		69(94.52)	35(89.74)	
Positive	6(5.08)	7(11.67)		4(5.48)	4(10.26)	

Data are presented as mean ± standard deviation or n (%). DIS, Distance from anal verge; Cir, Circumferential growth; MRF, Mesorectal fascia; EMVI, Extramural venous invasion; CEA, Carcinoembryonic antigen; CA199, Carbohydrate antigen 199.

### Radiomics model construction and comparison

The peritumoral 2 mm radiomics model failed to converge during development and thus could not be constructed. As a result, four radiomics models were ultimately developed: Habitat, INTRA, PERI1mm, and PERI3mm.

**Table 2** summarizes the diagnostic performance of the four radiomics models used to predict high-risk LARC. The SVM-based habitat model exhibited a notably high AUC of 0.934 in the training set; however, its performance declined to 0.713 in the test set, suggesting a potential risk of overfitting. Consequently, LR models were chosen as the basis for constructing radiomics signatures. The LR-based habitat model demonstrated superior AUC values compared to the intratumoral model (training: 0.882 vs. 0.752; test: 0.743 vs. 0.715). Similarly, the PERI3mm model outperformed the PERI1mm model (training: 0.820 vs. 0.794; test: 0.734 vs. 0.665).

**Table 2:** Diagnostic performance of radiomics models built with two classifiers.

Model	Classifier	Accuracy	AUC	Sensitivity	Specificity	Corhort
INTRA	LR	0.669	0.752	0.717	0.644	training
	LR	0.616	0.715	0.821	0.507	test
	SVM	0.803	0.819	0.733	0.839	training
	SVM	0.652	0.641	0.436	0.767	test
Habitat	LR	0.809	0.882	0.817	0.805	training
	LR	0.768	0.743	0.615	0.849	test
	SVM	0.860	0.941	0.950	0.814	training
	SVM	0.723	0.713	0.641	0.767	test
PERI1mm	LR	0.764	0.794	0.583	0.856	training
	LR	0.652	0.665	0.590	0.685	test
	SVM	0.860	0.858	0.683	0.949	training
	SVM	0.670	0.575	0.282	0.877	test
PERI3mm	LR	0.758	0.820	0.833	0.720	training
	LR	0.759	0.734	0.564	0.863	test
	SVM	0.837	0.934	0.900	0.805	training
	SVM	0.750	0.701	0.436	0.918	test

### Construction of the clinical model

Univariate regression analysis was performed on clinical features. Features with a significance level of P < 0.05 were subsequently included in multivariate regression analysis. Four clinical features were ultimately identified as significantly associated with high-risk rectal cancer: sex, age, depth, and mrN stage, with odds ratios (OR) of 0.43, 0.97, 1.22, and 2.53, respectively. These four features were used to construct the clinical prediction model.

### Construction of the integrated model (Nomogram)

**Figure 4:**
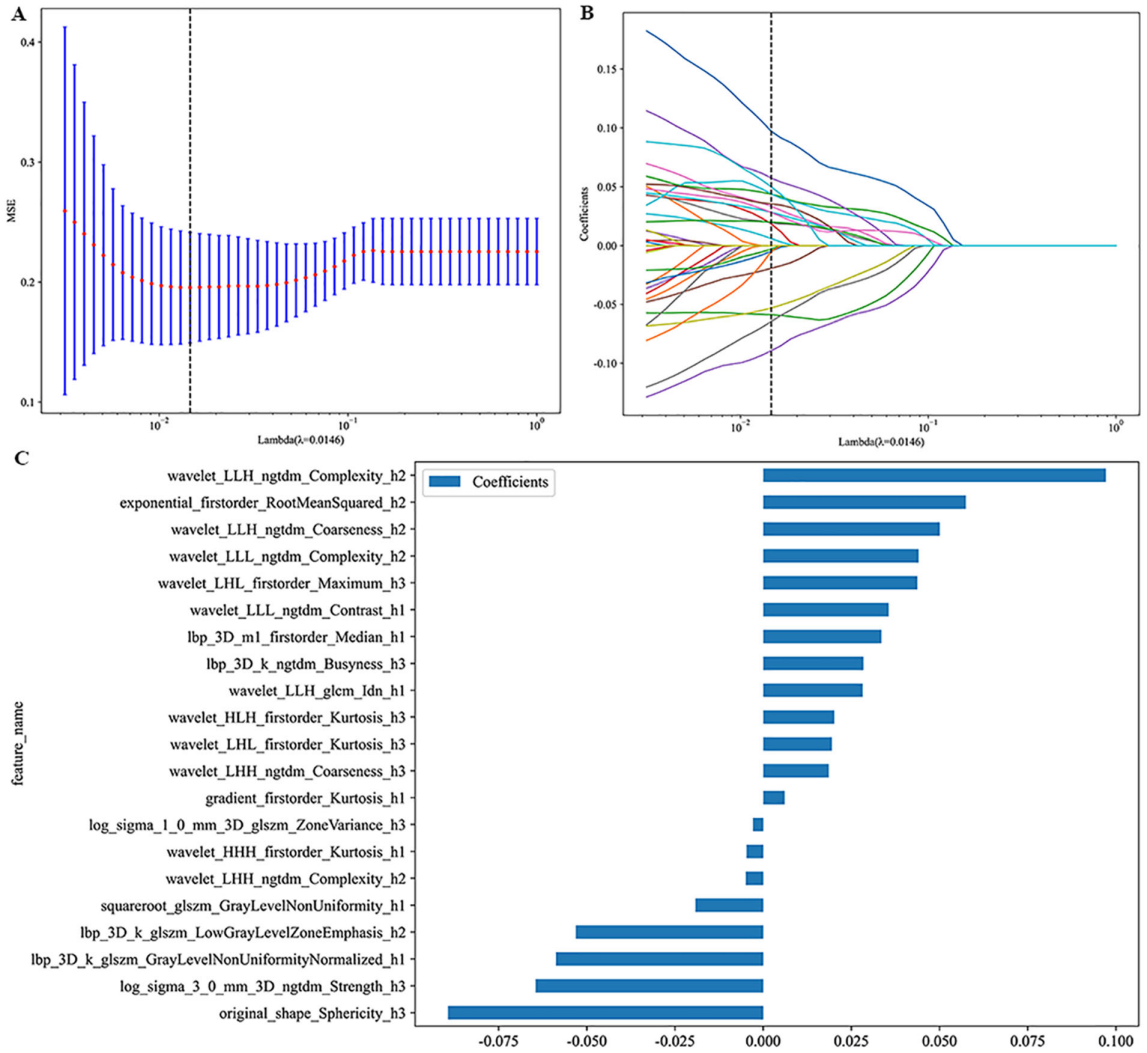
Radiomics feature selection based on LASSO of the habitat model. The MSE of LASSO regression **(A)**, the coefficients of cross-validation **(B)**, the weight coefficients of selected features **(C)**.

**Figure 5:**
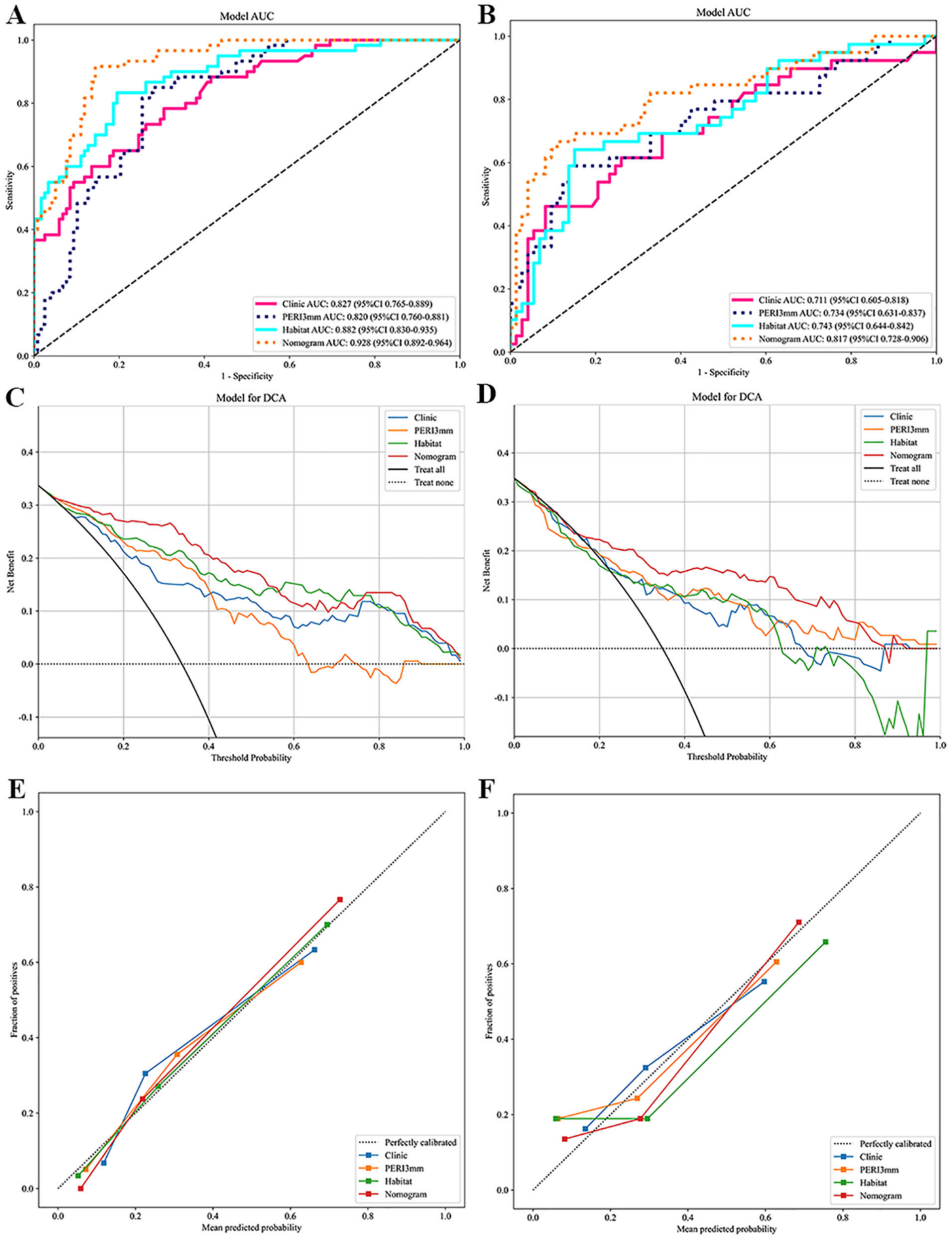
The performance of nomogram and other three models. The ROC, DCA and calibration curves of four models in the training **(A, C, E)** and test **(B, D, F)** corhorts. ROC, receiver operating characteristic; DCA, decision curve analysis.

The integrated model, which combined habitat features, PERI3mm features, and clinical features, exhibited the best predictive performance (**Table 3**). To improve model interpretability, a visual nomogram was developed (**Figure 4**). In both the training and test cohorts, the nomogram achieved significantly higher area under the curve (AUC) values (0.928 and 0.817, respectively) compared with all other individual models (**Figure 5**). The DCA curves showed that the nomogram offered the highest clinical net benefit across a broad range of threshold probabilities. Calibration curves revealed excellent concordance between the probabilities predicted by the nomogram and the observed proportions of high-risk LARC.

**Table 3:** Diagnostic performance of individual models and the integrated nomogram.

Signature	Accuracy	AUC	Sensitivity	Specificity	PPV	NPV	Cohort
Clinic	0.719	0.827	0.767	0.695	0.561	0.854	Training
Clinic	0.750	0.711	0.436	0.918	0.739	0.753	Test
PERI3mm	0.758	0.820	0.833	0.720	0.602	0.895	Training
PERI3mm	0.759	0.734	0.564	0.863	0.687	0.787	Test
Habitat	0.809	0.882	0.817	0.805	0.681	0.896	Training
Habitat	0.768	0.743	0.615	0.849	0.686	0.805	Test
Nomogram	0.871	0.928	0.900	0.856	0.761	0.944	Training
Nomogram	0.804	0.817	0.641	0.890	0.758	0.823	Test

AUC, Area under the curve; PPV, Positive predictive value; NPV, Negative predictive value.

## Discussion

This study developed a predictive model for high-risk LARC by integrating MRI habitat analysis, peritumoral radiomics, and clinical features. The nomogram demonstrated superior predictive performance in both the training cohort (AUC: 0.928) and the test cohort (AUC: 0.817), significantly outperforming models based on single features. These findings highlight the critical importance of tumor spatial heterogeneity and the peritumoral microenvironment in prognostic evaluation, offering novel imaging biomarkers for precise LARC stratification.

Conventional MRI plays a pivotal role in evaluating TNM staging and macroscopic features, such as MRF invasion; however, its capacity to resolve the TME is limited (15). In recent years, tumor habitat analysis has attracted growing interest. This method employs unsupervised clustering to analyze voxel-wise quantitative parameters, dividing tumors into distinct habitat subregions. Research has shown that habitat analysis, due to its superior ability to characterize tumor microenvironment heterogeneity (18, 21, 22), outperforms conventional radiomics in assessing tumor aggressiveness and prognosis. Highly aggressive habitat subregions may align with areas of immunosuppression or active angiogenesis, reflecting the metabolic traits of radiotherapy-resistant regions (23). Nevertheless, its application in predicting high-risk LARC remains underreported. Leveraging tumor microenvironment heterogeneity, this study utilized the K-means algorithm to segment lesions into three functional subregions and developed a habitat model by integrating intratumoral features. The results indicated that the model exhibited superior predictive performance compared to conventional radiomics, thereby addressing the homogeneity assumption inherent in traditional radiomics and more accurately reflecting the complexity of the TME. By incorporating multi-subregional parameters, the model more precisely captured tumor heterogeneity, consistent with prior research.

Habitat analysis offers three key interpretable advantages over conventional radiomics. First, it captures intratumoral heterogeneity by partitioning tumors into functionally distinct subregions (e.g., hypoxic, proliferative, and stromal compartments) based on voxel-wise radiomic features (19, 20). This subregional characterization reflects underlying tumor biology, as different habitats have been shown to correlate with distinct molecular pathways, immune infiltration patterns, and metabolic activities (21). Second, the spatial resolution of habitat features enables identification of aggressive subregions linked to treatment resistance and poor prognosis (22). Third, unlike conventional radiomics habitat analysis provides visualizable and interpretable subregions that can guide targeted biopsies, inform radiotherapy dose escalation to resistant subvolumes, and facilitate personalized treatment planning (24).

Radiomic features extracted from the 3-mm peritumoral region exhibited strong predictive power, suggesting that peritumoral stromal infiltration may influence prognosis by modulating tumor aggressiveness. This finding supports the mechanism proposed by Horvat et al. (13), in which peritumoral fibrosis facilitates local disease progression. Additionally, the integration of clinical features (e.g., mrN stage and tumor invasion depth) with radiomic features further improved model performance, aligning with the NCCN guideline recommendation for multiparametric evaluation in high-risk LARC (12).

This study innovates by constructing, for the first time, a multi-scale model for risk stratification of LARC based on MRI habitat analysis and peritumoral radiomics. Compared with traditional models, habitat analysis provides a more authentic representation of TME complexity by identifying functional subregions within the tumor. For example, highly invasive subregions may exhibit high levels of pro-angiogenic factors, such as vascular endothelial growth factor, which are linked to resistance to immunotherapy (25). Additionally, the predictive superiority of the 3-millimeter peritumoral region may stem from tumor-stroma interactions; similar studies in lung cancer have demonstrated that peritumoral texture features have independent prognostic value (26).

In clinical translation, this study offers critical evidence for the precise selection of candidates for TNT. Although the NCCN guidelines recommend TNT exclusively for high-risk patients, conventional staging methods may fail to detect occult high-risk cases and could inaccurately classify some low-risk patients as high-risk. Our model, which noninvasively identifies tumor heterogeneity, aids in distinguishing these patients, thereby preventing overtreatment of those at low risk. Additionally, habitat analysis has the potential to provide imaging-based evidence supporting the “watch-and-wait” approach, enabling personalized decision-making for organ preservation (27).

This study has several limitations. This study defined high-risk LARC based on postoperative pathology, while clinical application requires pre-treatment TNT selection—a limitation of the retrospective design. Although the pathological features used align with current risk stratification guidelines, future prospective studies are needed to validate the model’s ability to predict survival outcomes or treatment response, confirming its clinical utility for pre-treatment decision-making. The retrospective design may introduce selection bias, necessitating future prospective multicenter cohort studies to validate the model’s generalizability. Manual tumor segmentation introduces subjectivity, and incorporating deep learning techniques could enable automation. Multiparametric MRI sequences, such as DCE-MRI, were not included; future research should investigate their incremental diagnostic value.

In conclusion, this study demonstrates the potential value of MRI habitat analysis and the tumor microenvironment surrounding the tumor in risk stratification for LARC, providing imaging biomarkers for the transition towards a personalized treatment model. Future research should integrate multi-omics data (such as genomics and immune microenvironment) and artificial intelligence algorithms to further optimize the model and promote its clinical translation.

## Data Availability

The original contributions presented in the study are included in the article/**Supplementary Material**. Further inquiries can be directed to the corresponding authors.
