# Supplementary material for: Integrating MRI habitat heterogeneity and peritumoral radiomics into a nomogram for optimized risk stratification in locally advanced rectal cancer: a multicenter study
Source: Front Oncol. 2026 Apr 21;16:1739343. doi: 10.3389/fonc.2026.1739343 (PMC13138983; doi:10.3389/fonc.2026.1739343)
Supplement: Supplementary file 2 [file Table1.docx]

**Supplementary S1.** The main parameters of rectal MRI T2WI

| Hospital | MRI machine | Magnetic field strength | TR | TE | Slice thickness | Interval | FOV | Sequence |
| --- | --- | --- | --- | --- | --- | --- | --- | --- |
| Center 1 | Siemens Skyra | 3.0T | 6530 | 120 | 3 | 3.6 | 160×160 | TSE |
|  | GE Signa HDXT | 3.0T | 3240 | 107 | 4 | 4 | 180×180 | FSE |
| Center 1 | GE Discovery 750 | 3.0T | 7270 | 110 | 3 | 3.6 | 200×200 | FSE |
|  | Siemens Skyra | 3.0T | 5000 | 100 | 3 | 3.6 | 180×180 | TSE |
| Center 3 | GE Discovery 750 | 3.0T | 7270 | 110 | 3 | 3.6 | 200×200 | FSE |
|  | GE Signa HDXT | 3.0T | 4100 | 108 | 3.5 | 4 | 200×200 | FSE |
| Center 4 | GE Discovery 750 | 3.0T | 10187 | 66 | 5 | 1 | 280×280 | FSE |

Center 1, the Huangdao Hospital of the Affiliated Hospital of Qingdao University; Center 2, the Laoshan Hospital of the Affiliated Hospital of Qingdao University; Center 3, the Shinan Hospital of the Affiliated Hospital of Qingdao University; Center 4, Qingdao Municipal Hospital; TR, repetition time; TE, echo time; FOV, field of view

**Supplementary S2.** Features extracted from each voxel of the tumor VOI

firstorder:

- 'Entropy'

- 'MeanAbsoluteDeviation'

- 'Median'

glcm:

- 'DifferenceAverage'

- 'DifferenceEntropy'

- 'DifferenceVariance'

- 'JointEnergy'

- 'JointEntropy'

- 'InverseVariance'

- 'SumEntropy'

- 'Imc1'

- 'Imc2'

glrlm:

- 'RunEntropy'

- 'RunVariance'

- 'LongRunEmphasis'

glszm:

- 'SizeZoneNonUniformityNormalized'

- 'SmallAreaHighGrayLevelEmphasis'

ngtdm:

- 'Contrast'

- 'Strength'
